# 957. Impact of Preoperative Proton Pump Inhibitor Use on Skin Graft Complications and Scarring After Burns

**DOI:** 10.1093/jbcr/irag033.535

**Published:** 2026-04-06

**Authors:** Philong Nguyen, Joshua Wang, Yousef Tanas, Noelle Gonzalez, Kamryn K Baker, Sudhanvan Iyer, Cameron Bowers, Kelli Blackwell, Wei-Chen Lee, Juquan Song, Jong Lee

**Affiliations:** University of Texas Medical Branch, Galveston, TX; University of Texas Medical Branch, Galveston, TX; Houston Methodist Hospital, Houston, TX; University of Texas Medical Branch - John Sealy School of Medicine, Houston, TX; McGovern Medical School at University of Texas Health Science Center at Houston, Houston, TX; University of Texas Medical Branch, Galveston, TX; University of Texas Medical Branch, Galveston, TX; University of Texas Medical Branch, Galveston, TX; University of Texas Medical Branch, Galveston, TX; University of Texas Medical Branch, Galveston, TX; Division of Burn and Trauma Surgery, Department of Surgery, University of Texas Medical Branch, Galveston, Galveston, TX

## Abstract

**Introduction:**

Proton pump inhibitors (PPIs) are widely prescribed for gastrointestinal disorders and have been linked to impaired wound healing and increased infection risk in surgical populations. Burn patients frequently undergo skin grafting and are particularly vulnerable to complications, yet the influence of chronic PPI use on graft outcomes remains unclear. To date, no known study has specifically examined this relationship. This retrospective study evaluates the association between preoperative PPI use and skin graft-related complications after burn injury.

**Methods:**

Using the TriNetX Research Network, we identified adults ages 18 years and older with burn injuries who underwent split-thickness skin grafting (STSG) between 2016–2024 using ICD-10 and CPT codes. Chronic PPI use was defined as ≥3 prescriptions in the year before injury. Users were matched 1:1 with non-users using propensity scores based on demographics, body mass index (BMI), comorbidities, gastrointestinal disease, burn severity (total burn surface area burn), and STSG procedures. Primary outcomes included skin graft infection and failure. Secondary outcomes included hypertrophic scarring and fibrotic skin conditions. Outcomes were assessed at 30 and 60 days. Hazard ratios (HRs) with 95% confidence intervals (CIs) were calculated. Statistical significance was defined as log-rank *p*<.05.

**Results:**

The matched cohort included 2640 patients per group. PPI use was associated with higher rates of graft infection (30 days: 0.99% vs. 0.32%, HR 3.04, *p*=.004; 60 days: 1.41% vs. 0.73%, HR 1.97, *p*=.019), additional STSG procedures (30 days: 23.43% vs. 15.35%, HR 1.56, *p*<.0001; 60 days: 26.86% vs. 17.37%, HR 1.59, *p*<.0001), and fibrotic skin conditions(30 days: 8.58% vs. 5.66%, HR 1.55, *p*<.0001; 60 days: 14.10% vs. 10.72%, HR 1.36, *p*=.0002). No significant differences were observed in graft failure, unspecified graft complications, or hypertrophic scarring.

**Conclusions:**

Chronic preoperative PPI use is associated with increased risk of graft infection, increased grafting procedures, and scar fibrosis after burn injury. These findings underscore the importance of careful preoperative medication review and risk stratification. Prospective studies are needed to clarify mechanisms and guide interventions to optimize graft outcomes in burn patients.

**Applicability of Research to Practice:**

Recognizing PPI use as a potential contributor to graft complications and scarring may inform preoperative evaluation, surgical planning, and patient counseling to improve burn care outcomes.

**Funding for the study:**

This study was supported by foundation funding through the Institute for Translational Sciences (UL1 TR001439), funded by the National Center for Advancing Translational Sciences at the NIH.

